# Targeted Therapy Evolution from Defining a Sub-population to Crossing Multi-indications

**DOI:** 10.34172/apb.43306

**Published:** 2024-09-15

**Authors:** Daohong Chen

**Affiliations:** ^1^Research Institute, Changshan Biochemical Pharmaceutical, North Head of Yinchuan Street, Zhengding New District, Shijiazhuang, Hebei, China, 050800

**Keywords:** Targeted therapy, Drug repurpose, Tissue agnostic approval

## Abstract

**Purpose:**

It tends not only to shed lights on an emerging classification framework of disease according to the shared molecular pathogenesis across various organs/tissues, but also to inspire more efficient paradigms of pharmaceutic innovation in a broader medical perspective.

**Methods:**

Literature review and re-thinking.

**Results:**

This article has sorted out an updated profile of the outstanding targeted medications with an extending list of clinical indications in oncology and beyond.

**Conclusion:**

Pharmaceutic development can be processed in a less risky and more affordable manner through drug repurpose or tissue agnostic approval.

## Introduction

 During recent decades, taking the advantages of dramatic breakthroughs in biological science and technology the knowledge about disease etiology and pathogenesis has advanced significantly at molecular levels.^1,2^ As a result, numerous aspects of clinical medicine and pharmaceutical innovation have timely undergone a fundamental transformation from conventional treatment to targeted therapy.^2-4^ In contrast to traditional mode of one drug for the all patients with a classic type of illness, targeted therapy is designed to tackle the biological pathway(s) driving relevant pathological phenotypes, which can be differentially expressed among various sub-populations of same disease in the clinic.^3,4^ In corollary, with an unprecedented therapeutic efficacy and minimized adverse reactions compared to conventional medication, contemporary targeted therapy delivers clinical benefits precisely to a medical condition and even a subset of patients with a disease, particularly in oncology.^4,5^

 Given that a traditionally diagnosed neoplasm usually results from diverse molecular mechanisms known as heterogeneity of the pathogenesis, therapeutic strategy for various sub-groups of patients with a classic type of tumor may need distinct targeted approaches according to the expression of characteristic biomarker profiles.^1,5^ In this sense, each individual patient with a clinical diagnosis is advised to choose the right one of existing precise medications or a combination of several targeted agents depending upon the scenarios of altered disease-driven biological hallmark(s).^4^ Intriguingly in recent years, it has been increasingly demonstrated that a characteristic of molecular aberration behind a given pathology may be identified across multiple cancer types, instead of being limited to only one tumor category, to play a neoplastic promoting role.^6,7^ In light of this rationale, certain targeted compounds are corroborated to efficaciously tackle multiple clinical indications sharing a molecular mutation through the exceptionally efficient pharmaceutical development such as drug repurpose or tissue agnostic approval.^7,8^

 Targeted therapy has risen as a golden milestone in medical management of oncology since the earlier successes of the human epidermal growth factor receptor (EGFR)2 antibody and the breakpoint cluster region-Abelson (BCR-ABL) inhibitor for breast cancer and chronic myeloid leukemia respectively.^3,4^ Moreover, targeted medications are notably going beyond oncology to cover a broad variety of clinical areas, including autoimmunity, metabolic disorders, hematologic diseases, among others.^9,10^ In non-neoplastic fields, while there is a trend regarding the biomarker-based stratification of patient sub-populations with a disease, the scenarios of one targeted therapy across multiple indications are also emerging clinically.^9-11^ Thus to reflect the evolving dynamic of pharmaceutical development and medical care, this article presents a update on an array of highlighted therapeutic targets whose hitting compounds have the comprehensive efficacy of one drug for the multiple disease types. Insights herein are tending to inspire a highly efficient therapeutic innovation from the perspective of a shared/overlapped molecular pathogenesis in and beyond oncology.

## Human EGFR2 (HER2)

 Representing a member in the molecular family of EGFR tyrosine kinases, HER2 is composed of three sequential regions, namely extracellular, transmembrane, and cytoplasmic domains. Nonetheless, unlike the rest EGFR proteins, there are no cognate ligands for HER2 which needs to be dimerized with other members of this molecular family for initiating downstream signaling activities.^4^ The latter mediating pathways comprise rat sarcoma viral proto-oncogene/extracellular receptor kinase (RAS/ERK) and phosphoinositide 3-kinase/protein kinase B/mammalian target of rapamycin (PI3K/AKT/mTOR), to enhance cell proliferation and survival consequently.^4,12^ As a predictive biomarker and a therapeutic target, HER2 gene amplification and over-expression were identified in a spectrum of neoplastic types including breast cancer, gastric cancer, lung cancer, among others.^4,8^ Trastuzumab was the first HER2-targeted monoclonal antibody being approved to treat the biomarker positive breast cancer, based upon a phase III clinical trial showing to extend the progressing-free survival (PFS) and median overall survival (OS) by 2.8 months and 4.8 months respectively.^12,13^ Subsequently, the small chemical compounds such as lapatinib and neratinib were developed and approved for oral application in the targeted therapy of HER2 over-expressing breast cancer at advanced stages, through specifically binding to the tyrosine kinase site of the cytoplasmic domain and thus inhibiting downstream signaling activities.^12,14^ Besides, neratinib was highlighted to be exceptionally efficacious for tackling central nervous system metastasis of HER2 positive breast cancer.^15^ Recently, antibody-drug conjugate technique has emerged as an outstanding platform for developing a group of most complex biochemical compounds which combine excellent specificity of monoclonal antibodies and extremely potent chemical agents, to deliver higher efficacy than that of “naked” antibodies and much lower toxicity than that of traditional chemotherapy.^16^ In this light, trastuzumab emtansine (T-DM1) was constructed through linking trastuzumab with a tubulin disrupting-based chemical compound, and has been demonstrated to further extend PFS and OS of advanced HER2-expresing breast cancer surpassing the unconjugated antibody therapy.^17^ Moreover as the next generation product, trastuzumab deruxtecan (T-DXd) consists of a topoisomerase I inhibitor conjugated to trastuzumab by novel cleavable linker with improved efficiency, being able to out-perform T-DM1 in treatment of HER2-positive breast cancer in terms of PFS and OS improvement.^18^ Of note, upon expanding such superior clinical effectiveness beyond breast cancer, T-DXd has just been approved to manage several advanced neoplasms with HER2-overexpression/ mutation including gastric cancer and non-small lung cancer (NSLC).^12,19^ In addition, the therapeutic potentials of T-DXd are currently in on-going investigations in the patients with other tumors that exhibit HER2-expressing covering colorectal cancer, urothelial carcinoma, among others.^8,12^

## Tropomyosin receptor kinases (TRK)

 TRK proteins are encoded by the neurotrophic receptor tyrosine kinase (NTRK) genes, and principally expressed within the nervous system after embryogenesis, to facilitate regulation of pain, appetite, memory among other physiological events.^8,20^ Pathologically, the gene-associated chromosomal rearrangements have been identified across more than 20 distinct types of adult and pediatric malignancies.^21^ Regarding biological effects, these mutations lead to over-expression and phosphorylation of relevant TRK fusion proteins, which then constitutively activate down-stream signaling cascades such as ERK and PI3K to enhance cellular proliferation and survival.^20^ In corollary, the kinase domain activation upon TRK fusion has recently been validated as the molecular driver of the neoplastic pathogenesis, and can thus be exploited for pharmaceutical targeting.^10^ Intriguingly except a few types of rare neoplasms such as congenital ﬁbrosarcomas, TRK alterations occur in very low frequencies in common cancers of various organ/cellular origins.^21,22^ To address the unique challenge in contemporary medicine, an innovative trial design coupled with agnostic approval is emerging with an exceptional efficiency to develop the targeted therapy for combating numerous rare neoplasms and common tumor types with the rare genetic mutations.^6,20^ As the first approved chemical inhibitor against this target, larotrectinib was investigated through a novel basket trial study enrolling various malignant patients across 16 histological types bearing aberrant TRK activity, covering soft tissue sarcoma, breast cancer, lung cancer, among others.^8,22^ In this context, larotrectinib was corroborated to exert a highly efficacious outcome, namely an overall response rate of 80% and a median PFS of 9.9 months with an acceptable safety profile.^10,22^ Subsequently entrectinib, another kinase inhibitor against multiple targets, was authorized for human use to treat the NSCLC patient subgroup with c-ros oncogene1 (ROS1) aberration or numerous types of neoplasms with TRK fusion mutations.^23^ Whereas larotrectinib appeared superior over entrectinib in treatment of the cancers with TRK fusions based upon the comparable data of efficacy versus safety from relevant clinical trials, entrectinib has additionally been verified to pass through the blood-brain barrier and thus to be effective for tackling the metastatic brain tumors.^23,24^ Besides, to overcome the emerging challenge of acquired drug resistance resulting from recurrent kinase domain mutations induced by exposure to the first generation inhibitors, the TRK targeted compounds of next-generation such as selitrectinib (LOXO-195) have been accordingly discovered and developed through cell-based assays, animal models, and clinical studies.^25^

## Programmed cell death protein-1 (PD-1)

 As a hallmark of human immunity, T cells can differentially recognize exogenous versus endogenous antigens for the body, thus to initiate immune response or self tolerance respectively. This biological process is tightly controlled by an array of regulatory signaling pathways, in which PD-1 and its ligand PD-L1 serve a crucial checkpoint modulator to suppress the immune response.^26,27^ In the circumstances of neoplastic pathology, this negative regulating axis is intriguingly exploited to escape from immune surveillance upon over-expression of PD-1 or/and PD-L1 in tumor tissues.^26^ This contemporary immuno-oncology concept has notably inspired the pharmaceutic efforts to go beyond direct targeting malignant cell proliferation/survival previously, and instead to develop an exceptional therapy of blocking PD-1 signaling cascade for releasing the “molecular brake” on activated T lymphocytes against cancer which is known as immune checkpoint inhibitor (ICI).^28^ Since the first two monoclonal antibodies nivolumab and pembrolizumab were approved to treat advanced melanoma and NSCLC, the list of clinical oncology indications has been continuously extended across histological classification, which meanwhile shed a dramatic influence on pharmaceuticals to develop more following-up agents in this regard.^8,27^ Of note, the successes of several ICI antibodies have exemplified the high efficiency of adaptive clinical trials, particularly in the scenario of pembrolizumab that ran through a seamless phase I-II study enrolling 1235 patients in total to win the accelerated approval for two therapeutic indications and a companion diagnostic product.^10,29^ Interestingly, avelumab was authorized for the treatment of metastatic Merkel cell carcinoma based on an open label phase II trial which achieved a durable response in 28 (32%) of 88 patients plus 10% stable disease, which was assessed as being superior to the historic control of chemotherapy in literature.^30^ Regarding the safety, while ICI medication may induce immune-related adverse events (irAEs) which need to be carefully monitored, the antibodies against PD-1 and PD-L1 cause lower incidence of any grade irAEs than anti-cytotoxic T-lymphocyte antigen-4 (CTLA-4) antibodies do.^31^ Given that the efficacious rates of PD-1/PD-L1 antibodies are generally lower than 40% in patients with any tumor type of a defined histology, identification of predictive biomarkers for sensitive the sub-populations is becoming increasingly important.^27,28^ Impressively as a corroborated companion diagnostic, high microsatellite instability/deﬁcient mismatch repair (MSI-H/dMMR) have been linked to a higher tumor mutation burden, tumor neoantigen burden, T cell inﬁltration and PD-L1 expression in neoplastic tissues.^32^ Whereas several PD-1/PD-L1 antibodies were thereby approved for the treatment of locally spread colorectal cancer with MSI-H/dMMR expression, pembrolizumab represents the first tissue-agnostic medication authorized for use in patients with advanced solid tumors bearing this biomarker.^8,27^

## Janus kinase-signal transducer and activator of transcription (JAK-STAT)

 The JAK-STAT pathway represents an evolutionarily conserved molecular network that serves as an intracellular communication hub transmitting numerous signals from transmembrane receptors into the nuclei, thus to modulate a wide array of biological activities.^33,34^ Following binding with their cellular surface receptors, over 50 types of cytokines and growth factors are going through the JAK-STAT signaling pathways to play regulatory roles in immune response, hematopoiesis, cell differentiation, among others.^33^ Regarding relevant activities of JAK kinases, the four known members of the JAK protein family can preferably interact with cytoplasmic tails of distinct ligand receptors, which then induce corresponding STAT dimerization, nuclear translocation and transcriptional modulation, consequently to exert diverse biological functions.^35^ Whereas JAK1, JAK3 and tyrosine kinase (TYK)2 are principally activated by pro-inflammatory cytokines such as interleukin (IL)-6, IL-12 and interferons, JAK2 is primarily initiated by hematopoietic growth factors including erythropoietin, granulocyte macrophage colony-stimulating factor.^33,35^ In corollary, mutations or overexpression of JAK-STAT signaling components have been revealed to play a crucial role in the pathogenesis of a broad spectrum of human diseases covering autoimmune disorders and myeloproliferative neoplasms among others, and thus can be exploited as a therapeutic target for pharmaceutical development.^34,35^ Representing the first approved JAK-targeted compound for medical utilization, ruxolitinib acted as a JAK1 and JAK2 inhibitor that was demonstrated to alleviate symptoms, reduce spleen size and impressively improve OS of myeloﬁbrosis patients compared to those with placebo or traditional treatment in the clinical studies.^35^ Subsequently the application of ruxolitinib was also expanded to treat patients with polycythemia vera, another myeloproliferative neoplasm.^34^ On the other hand, coming up as a JAK1 and JAK3 inhibitor tofacitinib has been approved for management of patients with rheumatoid arthritis (RA) who were resistant or intolerant to conventional drugs such as methotrexate.^33^ Moreover, it was corroborated through multiple clinical investigations that tofacitinib conferred more efﬁcacious and safer outcomes than those of other disease-modifying anti-rheumatic drugs (DMARDs) in patients with RA.^33,36^ Likewise, therapeutic benefits of tofacitinib were then revealed in a number of other autoimmune diseases through clinical trials including induction and maintenance treatment in patients with moderately to severely active ulcerative colitis.^33,37^ In addition, another JAK1 and JAK2 inhibitor baricitinib has been first approved JAK-targeted agent to treat atopic dermatitis, while also being authorized for RA indication.^38^ Interestingly in recent years, a pan-JAK inhibitor peﬁcitinib appeared leading to a greater RA score improvement in the Asian ethnicity.^39^ Anyhow whereas the clinical indication list of JAK inhibitors is hopefully being extended through encouraging clinical studies to cover psoriasis, lupus erythematosus, transplant rejection among others, the potential adverse reactions such as increased opportunistic infections should not be ignored.^33-35^

## Low molecular weight heparin (LMWH)

 Heparin was discovered as a characteristic linear polysaccharide with anticoagulant activity, and has subsequently been applied in medical practice for decades to improve clinical outcomes in a number of serious pathological conditions.^40,41^ Regarding the mechanism of action, heparin agents bind to antithrombin III and form the complex triggering a conformational alteration, which then interact with thrombin and activated factor X (factor Xa) thus to inhibit downstream of the coagulant processing cascade.^41^ In corollary, heparin-based compounds have been primarily utilized for prevention and treatment of thrombotic events complicated in numerous medical conditions such as venous thromboembolism, coronary artery disease and extracorporeal circulation implementation.^40^ Given that unfractionated heparin (UFH) was developed as the preliminary product, the next generation agents have been derived from UFH through particular de-polymerization reactions and termed low molecular weight heparin (LMWH) covering nadroparin, dalteparin, enoxaparin as well as bemiparin.^41,42^ While UFH has a relatively shorter half-life time and is less dependent on renal excretion in terms of pharmacokinetics, LMWH medications confer more robust bio-availability, optimized anti-factor Xa/IIa activity ratios, and minimized risks of the drug-induced adverse events.^40,41^ To date, an array of heparin-based compounds including several UFH and LMWH products, has been accepted in World Health Organization’s (WHO;s) List of Essential Medicines.^40,42^ In contrast to various oral anticoagulants of small chemical molecules, LMWH agents appear not to pass through the placental barrier and neither to depend on hepatic metabolism, thus being preferable for pregnant patients or the combined therapy with potential drug-drug interactions, respectively.^40^ Interestingly in recent years, there is a trend for heparin to go beyond the traditional applications to tackle several off-labelled clinical indications.^42^ Due to their pleiotropic functions about clotting inhibition, endothelial protection and immune modulation, UFH and LMWH formulations were observed to remarkably improve the survival of patients with disseminated intravascular coagulation and to significantly reduce 28-day mortality of sepsis patients including those with.^40,43^ In parallel, heparin agents can orchestrate an additional set of biological effects covering anti-inﬂammation/anti-complement, trophoblast promotion and microvascular modulation, offering a rationale to address the unmet needs in pregnant clinic.^44^ While dramatically improving outcomes of in vitro fertilization, LMWH compounds were empirically utilized to certain early pregnancy complications and able to signiﬁcantly raise the live-birth rates (by 20%~30%) in patients suffering from recurrent miscarriage, particularly in those with antiphospholipid syndrome.^40,44^ Likewise, LMWH regimens have been demonstrated to improve respiratory function and to down-regulate inflammatory biomarkers in the field of pulmonary diseases including asthma, chronic obstructive pulmonary disease, among others; in this context, inhaled heparin was further revealed to minimize the systemic adverse reactions such as bleeding, without affecting the therapeutic effectiveness.^41,45^ Besides, while being able to alleviate plasma protein leaking into urine in the patients with diabetic nephropathy or nephrotic syndrome, LMWH agents were also shown to reduce the major complications and mortality of moderate and severe pancreatitis in the clinic.^40,41,46^

## Glucagon- like peptide- 1 (GLP- 1)

 As an endocrine peptide composed of 30 amino acids, GLP-1 is encoded by the pro-glucagon gene in L-cells of intestinal epithelium, to bind with a distinct receptor (GLP-1R) which belongs to the comprehensive signaling protein family of G- protein- coupled receptors.^47,48^ Due to wide expression of GLP-1R in multiple organs of human, upon the ligand binding this biological pathway orchestrates a great variety of physiological effects, namely enhancing pancreatic β-cell capacity, sensitizing periphery tissues to insulin, down-regulating glucagon production and appetite, among others.^48^ Naturally derived GLP-1 presents a short half-life time about 2 minutes in the body resulting from the rapid cleavage of enzyme dipeptidylpeptidase-4 (DPP-4) and renal clearance of low molecular weight peptides.^48,49^ To circumvent the challenge posed by immediate diminishment of this native peptide in circulation, considerable pharmaceutical efforts have been made to prolong the half-life time through designing and developing structural variants of GLP-1R ligands with optimized pharmacokinetic (PK) trajectories.^48^ Representing the first GLP-1R agonist medication approved to treat type-2 diabetes (T2D), exenatide was derived from a Gila monster-secreted peptide that has 50% sequence homology with native human GLP-1, and administered via subcutaneously twice daily base on its half-life time 2-4 hours due to insensitivity to DPP-4.^50^ Subsequently, a human GLP-1 analogue agent liraglutide holding a half-life time of 13 h was created through the peptide sequence position 34 mutation and position 26 attached with a fatty acid that reversibly binds to plasma albumin, thus being resistant to DPP-4 cleavage and glomerular filtration.^50,51^ In comparison with exenatide during the clinical trials, liraglutide once daily appeared to confer a higher therapeutic efficacy on glycated hemoglobin (HbA1c) control, while inducing lower incidence of anti-drug antibody owing to 97% homology with native human GLP-1.^50^ Moreover to upgrade the PK profile through further structural modifications, semaglutide came up with a half-life time of 48 h and is administered once weekly, leading to greater weight loss and fewer drug discontinuations due to adverse events compared to other GLP-1 agonists in the clinic.^50,52^ Recently as a co-agonist of dual GLP-1 and glucose-dependent insulinotropic polypeptide receptors, tirzepatide has been authorized to manage T2D, showing even a higher efficacy than semaglutide in regard of HbA1c reduction and body weight control.^52,53^ Besides the classic indications to treat T2D and obesity, clinical applications of GLP-1 agonists are currently running into a dramatic phase of expansion, of which cardiovascular (CV) benefits have been highlighted to lower the risks of CV death, stroke and heart failure.^54^ While contributing to kidney protection upon reducing macroalbuminuria^54^ GLP-1 agents are notably corroborated to diminish liver fat content, inflammation, and fibrosis in patients with non-alcoholic steatohepatitis.^48,49^ In addition, likely resulting from their neurotrophic property and neuroprotective potential, GLP-1 compounds were observed to improve cognitive and motor assessment scores during clinical studies of neurodegenerative disorders such as Alzheimer’s disease Parkinson’s disease.^48^

## Perspective

 During recent two decades, rise of targeted therapy has revolutionized the landscapes of medical care and pharmaceutical development (Table 1), to confer the unprecedented efficacy with minimized toxicity versus traditional compounds, particularly in oncology.^3,4^ As a necessary tool for successful implementing targeted medicine strategy, biomarkers are cumulatively identified taking advantages of the breakthroughs from contemporary disease biology.^5,55^ Initially biomarkers were utilized to define the drug-sensitive subgroup of patients and thus to avoid in-efficacious treatment on others, as being exemplified by the scenario of HER2 for breast cancer management.^12^ Of note, while having dramatically advanced our knowledge about etiology and pathogenesis of numerous medical conditions, the co-evolution of biomarkers and targeted therapy continuously contribute to the expansion of clinical applications upon drug repurpose following the first approval.^7,10^ The repurposing approaches can lead to less risky and more affordable pharmaceutical achievements through fast development; they are inspired by a scientific rationale of shared biological mechanisms between certain clinical disorders of various organs/tissues, and may thus skip many non-clinical studies as well as phase I trials that have been performed for the first indication.^7^ Recently the advent of tissue agnostic drug approval is coming up with an even more efficient mode of therapeutic innovation since the successes of TRK inhibitors and PD-1 antibodies to treat relevant biomarker-positive neoplasms across a broad spectrum of organs/tissues.^6,8^ In this light, whereas there is an emerging concept to advance the classifying principles of neoplasms according to common molecular pathogenesis-driven pathways regardless of their organ/tissue origins,^56^ exceptional impacts of local factors on therapeutic responses should not be ignored such as managing the v-Raf murine sarcoma viral oncogene homolog B1 (BRAF) mutated patients with colon cancer.^8^

**Table 1:** Representative targeted therapy for multiple indications

**Application**	**Target**	**Medication**	**Indication**	**Reference**
Oncology	HER2	Trastuzumab	-	^12,13^
		Lapatinib	HER2 + breast cancer	^12,14^
		Neratinib	-	^15^
		T-DM1	-	^17^
		T-DXd	HER2 + breast cancer, GC, NSCLC	^12,19^
	TRK	Larotrectinib	Multiple NTRK + tumors	^10,22^
		Entrectinib	Multiple NTRK + tumors, ROS1 + NSCLC	^23,24^
	PD-1	Nivolumab	Melanoma, NSCLC	-
		Pembrolizumab	MSI-H/dMMR + colorectal cancer	^8,27^
		Avelumab	Merkel cell carcinoma	^30^
Non-neoplasm	JAK-STAT	Ruxolitinib	Myeloﬁbrosis, polycythemia vera	^34,35^
		Tofacitinib	RA, ulcerative colitis	^33,36,37^
		Baricitinib	RA, atopic dermatitis	^38^
		Peﬁcitinib	RA	^39^
	Factor Xa/IIa	Nadroparin	Venous thromboembolism,	-
		Dalteparin	coronary artery disease,	^40-44^
		Enoxaparin	extracorporeal circulation, COVID-19	-
	GLP-1R	Exenatide	Type 2 diabetes,	-
		Liraglutide	obesity,	^50-54^
		Semaglutide	cardiovascular complications	-
		Tirzepatide	-	-

 Currently, the interesting phenomena of precision medicine tackling one sub-group of patients versus crossing multiple types of diseases are going beyond oncology into autoimmunity, hematology, metabolic disorders, among others.^33,40,54^ Whereas JAK inhibitors appear exerting higher efficacy in the interferon signature biomarker-responsive patient sub-set,^57^ various autoimmune disorders often share certain biological hallmarks such as autoantibodies.^58^ Although aberrant coagulating cascade activities may be complicated in numerous severe medical conditions,^40^ clinical outcomes upon heparin agent management depend on the core mediators including the expressing levels of heparin-bind protein and factor VIII.^59^ While GLP-1R agonists are conferring therapeutic benefits to an extending list of chronic illness,^48^ the clinical effectiveness is affected by specific gene alterations of GLP-1R and transcriptional factor 7-like 2.^60^ Overall, to date the clinical indication expansion of targeted therapy in non-neoplastic fields is primarily through drug repurposing processes,^10,40,48^ yet without successful examples of tissue agnostic approval probably until more robust biomarkers being validated. Anyway, targeted therapy has enjoyed an evolving journey from defining inter-patient heterogeneity to tackling multiple indications; the dialectic insights herein not only shed lights on an exceptional classification framework of disease according to the common mechanisms of molecular pathogenesis across various organs/tissues, but also inspire more efficient working paradigms of pharmaceutic innovation for superior medication to address unmet clinical needs.^5,55^

## Conclusion

 Pharmaceutic development can be processed in a less risky and more affordable manner through drug repurpose or tissue agnostic approval.

## Competing Interests

 None declared.

## Ethical Approval

 Not applicable.
